# Emotion recognition deficits in children and adolescents with autism spectrum disorder: a comprehensive meta-analysis of accuracy and response time

**DOI:** 10.3389/frcha.2024.1520854

**Published:** 2025-01-14

**Authors:** Maryam Masoomi, Mahdieh Saeidi, Rommy Cedeno, Zahra Shahrivar, Mehdi Tehrani-Doost, Zerimar Ramirez, Divya Aishwarya Gandi, Sasidhar Gunturu

**Affiliations:** ^1^ Department of Psychiatry, School of Medicine, Tehran University of Medical Sciences, Tehran, Alborz, Iran; ^2^ Department of Psychiatry, BronxCare Health System, New York, NY, United States; ^3^ Department of Psychiatry, Columbia University, New York, NY, United States; ^4^ Division of Molecular Therapeutics, Department of Psychiatry, New York State Psychiatric Institute, New York, NY, United States; ^5^ Research Center for Cognitive and Behavioral Sciences, Roozbeh Psychiatry Hospital, Tehran University of Medical Sciences, Tehran, Iran; ^6^ Medical School, Maharaja Sayajirao University of Baroda, Vadodara, India; ^7^ Bronx-Lebanon Hospital Center, New York, NY, United States; ^8^ Department of Psychiatry, Icahn School of Medicine at Mount Sinai, New York, NY, United States

**Keywords:** autism spectrum disorder, emotion recognition, meta-analysis, response time, social cognition

## Abstract

**Background:**

Autism spectrum disorder is a neurodevelopmental condition characterized by persistent challenges in social communication and restricted, repetitive behaviors. Emotion recognition deficits are a core feature of ASD, impairing social functioning and quality of life. This meta-analysis evaluates emotion recognition accuracy and response time in individuals with autism spectrum disorder compared to neurotypical individuals and those with other neurodevelopmental disorders.

**Methods:**

This systematic review with a meta-analysis was conducted following PRISMA guidelines. A comprehensive literature search across PubMed, Scopus, Cochrane Library, and Web of Science identified 13 studies published between 2006 and 2024. Data on emotion recognition accuracy and response times were synthesized using standardized mean differences in random-effects models. Heterogeneity was assessed using the *I*^2^ statistic, and sensitivity analyses were performed to ensure robustness.

**Results:**

Individuals with ASD exhibited significantly lower overall emotion recognition accuracy compared to TD individuals (SMD = −1.29, 95% CI: −2.20 to −0.39, *p* < 0.01) and NDDs (SMD = −0.89, 95% CI: −1.23 to −0.55, *p* = 0.02). Response times were significantly prolonged in ASD compared to TD individuals (SMD = 0.50, 95% CI: 0.36–0.63, *p* < 0.01) but not when compared to NDDs. Emotion-specific analyses did not consistently reveal significant differences across emotions (fear, anger, happiness, sadness, disgust, surprise), with substantial heterogeneity observed across studies (*I*^2^ > 50%).

**Conclusions:**

This systematic review with a meta-analysis highlights significant impairments in emotion recognition accuracy and processing speed among individuals with autism spectrum disorder, particularly compared to neurotypical individuals. These findings underscore the importance of developing targeted interventions to address these deficits, which are foundational to improving social cognition and quality of life in autism spectrum disorder. Future research should prioritize standardized methodologies and explore cultural and contextual factors influencing emotion recognition abilities.

**Systematic Review Registration:**

https://www.crd.york.ac.uk/PROSPERO/display_record.php?RecordID=627339, PROSPERO (CRD42024627339).

## Introduction

Autism Spectrum Disorder (ASD) is a complex neurodevelopmental condition characterized by persistent challenges in social communication and restricted, repetitive patterns of behavior (1). These difficulties significantly impair the ability to interpret and respond to social cues, such as facial expressions, vocal tone, and body language, which are critical for successful social interactions and relationship-building (2, 3). Recent data suggest that ASD affects approximately 1 in 36 children in the United States, with boys being four times more likely than girls to receive a diagnosis (4).

Emotion recognition deficits are among the most significant challenges faced by individuals with ASD, contributing to impaired social functioning and diminished quality of life (5, 6). Research consistently shows that children and adolescents with ASD struggle to recognize subtle or complex emotions, such as sadness or fear, more than basic emotions like happiness (7–9). These deficits extend beyond accuracy to include slower response times, reflecting inefficiencies in cognitive processing (10, 11). Comparisons with neurotypical individuals consistently demonstrate these differences. For instance, children with ASD tend to focus less on the eye region of faces—a key source of emotional information—and more on less informative areas (12, 13). Multimodal research confirms atypical patterns in eye gaze and facial emotion processing, underscoring the interplay between visual attention and emotional interpretation (14).

The literature highlights distinct behavioral and cognitive profiles between ASD and neurotypical children. For example, while neurotypical children rely on holistic face processing strategies, individuals with ASD often exhibit feature-based processing, which hinders accurate emotion recognition, particularly for negative emotions such as fear and sadness (15, 16). These differences extend to response times, with children with ASD showing prolonged reaction times during emotion recognition tasks compared to their peers, likely reflecting underlying cognitive inefficiencies (6, 17).

Emotion recognition deficits, often tied to broader emotion regulation challenges, are foundational to understanding social impairments in ASD (18). Neuroimaging studies further distinguish individuals with ASD from neurotypical individuals by revealing atypical activation patterns in key emotion processing regions such as the amygdala and fusiform gyrus (13, 19). These findings suggest that ASD-related deficits in emotion recognition arise from disruptions in both social and neural pathways, contributing to pervasive social communication challenges.

Prior meta-analyses have provided valuable insights into emotion recognition deficits in ASD, but limitations remain. For example, reviews by Harms et al. and Uljarevic and Hamilton primarily focus on accuracy while neglecting response time and emotion-specific variability (5, 6). Additionally, heterogeneity in methodologies, task designs, and participant characteristics complicates the interpretation of findings (7, 9). More recent reviews (20, 21) underscore these methodological gaps, particularly the need for studies addressing contextual and cultural factors that influence emotion recognition outcomes. These heterogeneities are further compounded by modality-specific differences in emotion recognition, with evidence suggesting variability across visual and auditory domains (21).

Recent advancements in sensing technologies and machine learning offer promising avenues for improving emotion recognition assessments in ASD (22).

This study also considers how factors such as task complexity, age, and cultural differences may contribute to variability in findings, as highlighted in recent literature (21, 23). While these factors are not directly tested in this meta-analysis, they are discussed as potential moderators influencing the observed heterogeneity.

This meta-analysis aims to address these gaps by systematically evaluating emotion recognition accuracy and response time in children and adolescents with ASD compared to neurotypical individuals and individuals with other neurodevelopmental conditions. By incorporating response time as an outcome measure and analyzing emotion-specific variability, this study seeks to provide a more nuanced understanding of the cognitive and behavioral mechanisms underlying emotion recognition deficits in ASD. Specifically, this analysis addresses the following research question: How do children and adolescents with ASD differ from neurotypical individuals and individuals with other neurodevelopmental conditions in emotion recognition accuracy and response time?

### Objective

#### General objective

To systematically evaluate emotion recognition deficits, including accuracy and response times, in individuals with ASD compared to neurotypical individuals and those with other neurodevelopmental disorders.

#### Specific objectives

To assess differences in emotion recognition accuracy and response times between individuals with ASD and neurotypical individuals and those with other neurodevelopmental disorders.

To investigate whether deficits in emotion recognition are consistent across specific emotions (happiness, sadness, anger, fear, surprise, disgust) in individuals with ASD.

## Methods

### Study design and reporting

This systematic review with a meta-analysis was conducted in accordance with the Preferred Reporting Items for Systematic Reviews and Meta-Analyses (PRISMA) guidelines to ensure transparent and replicable methodology (24).

### Literature search

A comprehensive literature search was conducted across four major electronic databases: PubMed, Scopus, Cochrane Library, and Web of Science (WOS), including all relevant studies published up to September 2024. The search strategy utilized a combination of keywords and Medical Subject Headings (MeSH) terms related to autism spectrum disorder (ASD), emotion recognition, and neurodevelopmental assessments to ensure broad coverage of pertinent studies. Reference lists of included studies and relevant reviews were also manually screened to identify additional eligible studies.

The search terms were combined using Boolean operators (“AND/OR”) to refine the database queries. For example, the following search string was applied in PubMed: (“emotion recognition” OR “facial expression recognition” OR “emotion processing” OR “social cognition”) AND (“autism spectrum disorder” OR ASD OR autism OR “neurodevelopmental disorders”). Database-specific subject headings were used for searches in Scopus, Cochrane, and Web of Science, with keywords such as “autism spectrum disorder,” “facial expression recognition,” “emotion recognition,” “emotion processing,” and “social cognition.” The search was limited to studies published between 2006 and 2024, and only English-language publications were included.

This systematic review with a meta-analysis has been registered with PROSPERO (ID: CRD42024627339). The final search was conducted on December 12, 2024, across all databases and PROSPERO.

### Eligibility criteria

#### Inclusion criteria

In this study, the criteria for selecting documents for the systematic review with a meta-analysis were based on the PICOS framework (Population, Intervention/Exposure, Comparator, Outcomes, Study design). The eligibility criteria included the following conditions:

• **Population**: Individuals diagnosed with ASD based on standardized criteria (DSM-III, DSM-IV, DSM-5, or ICD-10).
• **Comparator**: Neurotypical individuals or those with other neurodevelopmental disorders [e.g., ADHD (Attention-Deficit/Hyperactivity Disorder), intellectual disabilities, Prader-Willi syndrome].
• **Outcomes**: Studies reporting emotion recognition accuracy and/or response times as primary outcomes measured using standardized assessment tools.
• **Study Design**: Observational or experimental studies with at least 15 participants in both ASD and comparator groups.
• **Setting**: Studies conducted in any geographic region were included.
• **Language**: Only English-language publications were included. Articles in other languages were considered only if a reliable translation was available.
• **Publication Year**: Articles published between January 2006 and September 2024 were included.

### Exclusion criteria

Articles were excluded if they:

• Did not provide sufficient data to calculate effect sizes for emotion recognition accuracy or response times.
• Focused on populations with unspecified comorbid conditions.
• Were reviews, editorials, conference abstracts, or unpublished dissertations.
• Did not include a comparator group.

The titles and abstracts of all retrieved articles were screened using the prespecified inclusion and exclusion criteria before the retrieval of full-text articles for further screening. Two reviewers independently performed the initial screening. In the second step, the two reviewers independently read the full texts of articles that were not excluded in the initial stage and assessed their eligibility based on the inclusion criteria. Discrepancies between the two reviewers were resolved by consensus or through consultation with a third reviewer.

### Data extraction

The study selection process was managed using EndNote Desktop version 20.2.1 (25) to organize and remove duplicate records. The initial screening of titles and abstracts was conducted using Rayyan web-based software (26), facilitating the identification of relevant studies. Two medical doctors as independent reviewers meticulously evaluated the studies in two stages: first, by screening titles and abstracts for relevance and subsequently by conducting a full-text review to determine eligibility based on the inclusion and exclusion criteria. Any discrepancies between the reviewers were resolved through discussion or consultation with a third reviewer to ensure consistency and accuracy in the selection process. This procedure is visually represented in the PRISMA flowchart.

Data was systematically extracted from each study using a standardized form to ensure consistency and comprehensiveness. Extracted data encompassed study characteristics such as author(s), year of publication, country of study, and participant details including sample size, age range, sex distribution, intellectual abilities, and comorbidities. Methodological information, including the study design, diagnostic criteria for ASD, types of control groups, and emotion recognition assessment tools, was also recorded. Outcome measures focused on emotion recognition accuracy and response time, with means, standard deviations, and sample sizes for both the ASD and control groups meticulously documented.

### Quality assessment

The methodological quality of the included studies was independently assessed by two reviewers using the Newcastle-Ottawa Scale (NOS) for non-randomized studies (27). NOS evaluates studies across three domains: the selection of study groups, the comparability of groups, and the ascertainment of outcomes. Each study could receive up to nine stars, with higher scores reflecting better methodological quality.

Inter-rater reliability for study selection and quality assessment was calculated using Cohen's kappa statistics. Two independent reviewers screened titles and abstracts, as well as full-text articles, and assessed the quality of included studies. Discrepancies were resolved through consensus or consultation with a third reviewer. The inter-rater agreement for the initial title and abstract screening was substantial, with Cohen's kappa *κ* = 0.82. For full-text screening, the agreement was almost perfect (*κ* = 0.85). The inter-rater agreement for the quality assessment was *κ* = 0.80, indicating substantial agreement. The quality scores for all included studies are detailed in the accompanying tables.

### Statistical analysis

Statistical analyses were conducted using R version 4.4.2, employing the “metafor' package to perform the meta-analyses. Effect sizes were calculated as standardized mean differences (SMD) with 95% confidence intervals (CIs) for continuous outcomes, specifically, emotion recognition accuracy and response time. A random-effects model was used to account for between-study variability. Meta-analyses were performed to assess overall emotion recognition accuracy, as well as emotion-specific recognition for emotions such as fear, anger, disgust, happiness, sadness, and surprise. Response time analyses were conducted for these specific emotions.

Heterogeneity across studies was evaluated using the *I*^2^ statistic and tau-squared (*τ*^2^) values, with an *I*^2^ value exceeding 50% indicating substantial heterogeneity. When significant heterogeneity was detected, sensitivity analyses were performed by excluding studies that contributed disproportionately to the heterogeneity to assess the robustness of the findings.

### Study selection

**Figure 1:**
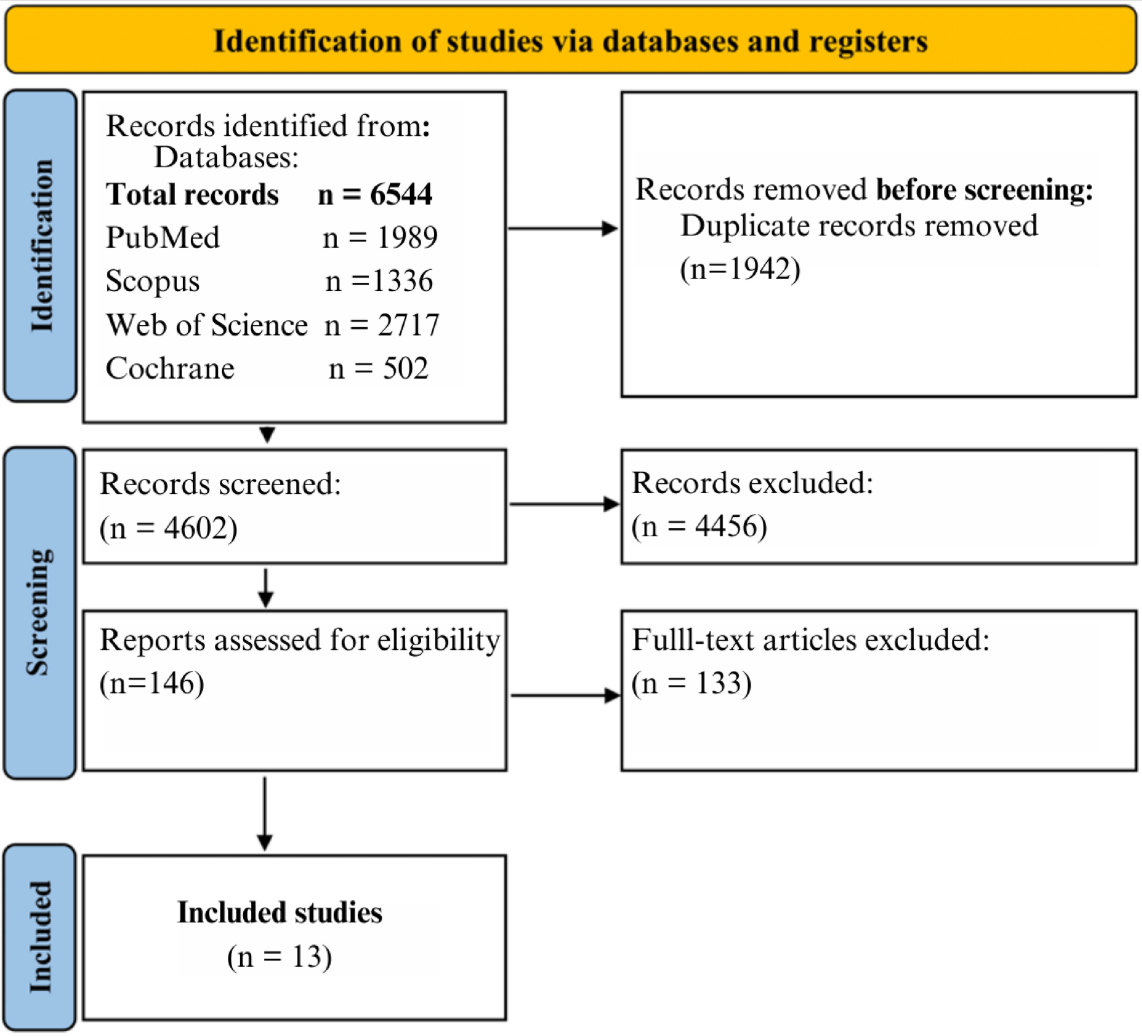
PRISMA flowchart illustrating study selection.

A total of 13 (28–40) studies were included in this meta-analysis. The selection process is detailed in the PRISMA flowchart (Figure 1), which illustrates the identification, screening, and eligibility stages. The initial search of multiple databases yielded 6,544 records. After removing duplicates and screening the titles and abstracts, 146 full-text articles were assessed for eligibility. Of these, 125 studies were excluded because they did not meet the inclusion criteria, leaving 13 studies in the final analysis.

## Results

### Study characteristics

The included studies (Table 1) spanned 2006–2024 and represented a diverse set of countries, including Spain, Germany, Korea, Italy, and the USA. The sample sizes ranged from 15 to 71 participants, all diagnosed with ASD, and compared to control groups (healthy controls or individuals with other neurodevelopmental conditions, such as Prader-Willi syndrome, ADHD, or intellectual disabilities). The participants' ages ranged from 6 to 18 years, with various intellectual abilities and comorbidities reported across studies.

**Table 1:** Characteristics of included studies.

Study ID	Design	Country	Study arms	Sample size	Population	Tools/(Mean ± SD)	Conclusion	Study aims
Perosanz et al. (28)	Prospective cohort	Spain	ASD 15, PWS 15, HC 15	15	Children (9–12 years)	**FEEL** Response Accuracy (Mean ± SD): Control: 30.95 ± 5.27 PWS: 21.95 ± 7.35 ASD: 4.33 ± 2.41 Reaction Time (Mean ± SD, ms): Control: 3,382.61 ± 1,520.23 PWS: 9,950.83 ± 11,254.90 ASD: 3,684.10 ± 1,954.78	ASD lower emotional accuracy	Comparing emotion recognition accuracy between ASD, Prader-Willi Syndrome (PWS), and healthy controls (HC).
Nagy et al. (29)	Prospective cohort	UK	ASD 9, HC 9	9	Adolescents (12–17 years)	FER task Response Accuracy (%): Timed: ASD 66.5 ± 11.7, Non-ASD 75.3 ± 9.1 Non-Timed: ASD 70.4 ± 10.5, Non-ASD 77.6 ± 8.4 Reaction Time (ms): Timed: ASD 2,102 ± 491, Non-ASD 1,835 ± 433 Non-Timed: ASD 2,543 ± 673, Non-ASD 2,321 ± 562	Atypical facial emotion processing	To analyze atypical facial emotion processing in adolescents with ASD.
Greco et al. (30)	Prospective cohort	Italy	ASD 20, ADHD 21, HC 21	20	Children (7–12 years)	Morphing task Happiness Recognition: ADHD: 65.7 ± 12.1 ASD: 57.3 ± 14.4 TD: 78.4 ± 10.2 Anger Recognition: ADHD: 52.4 ± 14.2 ASD: 49.6 ± 15.3 TD: 68.9 ± 12.5 Fear Recognition: ADHD: 47.8 ± 15.6 ASD: 45.2 ± 14.8 TD: 62.3 ± 11.4 Sadness Recognition: ADHD: 58.9 ± 11.5 ASD: 50.7 ± 13.9 TD: 70.1 ± 9.6	Clinical implications	To investigate clinical implications of emotion recognition differences between ASD, ADHD, and HC.
Hauschild et al. (31)	Prospective cohort	USA	ASD 52, HC 40	52	Adolescents (11–14 years)	Facial Emotion Recognition ASD Group: Adult Faces: Mean = 72.76%, SD = 11.08 Child Faces: Mean = 81.41%, SD = 12.66 For the Non-ASD Group: Adult Faces: Mean = 75.10%, SD = 12.39 Child Faces: Mean = 83.54%, SD = 11.40	FER performance for child faces	To evaluate performance differences in facial emotion recognition of child faces in ASD.
Mazzoni et al. (32)	Prospective cohort	Italy	ASD 25, ASD-ID 17, HC 27	25	Mean age 9.88	Emotion recognition task HFA: Fear (19.31, 21.02), Happiness (15.16, 16.49), Neutral (20.77, 16.70). LFA: Fear (21.78, 23.31), Happiness (20.35, 16.60), Neutral (20.63, 20.15). TD: Fear (9.86, 10.80), Happiness (8.63, 13.33), Neutral (7.08, 10.32)	Accuracy improved in ASD without ID	To compare accuracy improvements in emotion recognition in ASD with and without intellectual disabilities.
He et al. (33)	Prospective cohort	China	ASD 21, HC 21	21	Children (6–11 years)	Facial Emotion Expression FDT: ASD group: M = 667.19, SD = 42.89 TD group: M = 839.12, SD = 38.51 FC: ASD group: M = 1.72, SD = 0.14 TD group: M = 2.15, SD = 0.12	Emotion recognition deficits	To study emotion recognition deficits in children with ASD.
Liu et al. (34)	Prospective cohort	Taiwan	ASD 71, HC 63	71	Mean age 14.37	FERT FERT 1: ASD = 13.80, Non-ASD = 7.10 FERT 2: ASD = 17.95, Non-ASD = 9.13 FERT 3: ASD = 20.92, Non-ASD = 21.36	Subtle facial emotion deficits	To analyze subtle facial emotion recognition deficits in adolescents with ASD.
Kuusikko-Gauffin et al. (35)	Prospective cohort	Finland	ASD 34, HC 34	34	Mean age 12.5	FEFA Egyptian ASD: Mean = 24.4, SD = 3.3 Finnish ASD: Mean = 27.0, SD = 3.4 Egyptian TD: Mean = 31.4, SD = 3.3 Finnish TD: Mean = 30.7, SD = 3.4	Cultural impact on emotion recognition	To assess cultural impacts on emotion recognition in ASD.
Høyland et al. (40)	Prospective cohort	Norway	ASD 49, HC 49	49	Adolescents (12–21 years)	Visual Continuous Test ASD: Mean = 338.3 ms, SD = 65.0 ms TD: Mean = 330.5 ms, SD = 62.0 ms	Longer reaction times in ASD	To examine prolonged reaction times for emotion recognition in ASD.
Xavier et al. (36)	Prospective cohort	France	ASD 19, HC 19	19	Mean age 9.95	Emotion recognition Bimodal Task: ASD > 70% accuracy, better than unimodal tasks. Joy: ∼90% accuracy (easiest emotion). Neutral & Anger: < 50% accuracy (most difficult). Developmental Age: Positively associated with performance, especially in TD and ASD bimodal tasks. Visual vs. Bimodal: ASD performed worse on visual tasks but improved with bimodal cues.	Multimodal task support	To investigate multimodal support for emotion recognition in ASD.
Tell et al. (37)	Prospective cohort	USA	ASD 17, HC 17	17	Children (8–12 years)	Emotion Recognition Task TD: Happy: Direct 88.0 (3.6), Averted 92.8 (4.4) Sad: Direct 72.0 (7.2), Averted 78.7 (7.2) Angry: Direct 78.0 (4.8), Averted 96.3 (4.3) Fear: Direct 88.1 (6.6), Averted 74.7 (6.0) ASD: Happy: Direct 94.8 (3.6), Averted 89.6 (4.4) Sad: Direct 54.1 (6.4), Averted 68.5 (7.1) Angry: Direct 77.5 (4.2), Averted 86.2 (4.7) Fear: Direct 54.9 (5.6), Averted 54.5 (6.0)	Gaze direction impacts emotion perception	To explore the impact of gaze direction on emotion perception in ASD.
Rump et al. (38)	Prospective cohort	USA	ASD 19, HC 18	19	Mean age 6.4	Development of Emotion ASD: Mean = 1.80, SD = 0.52 TD: Mean = 2.42, SD = 0.59	Young ASD children can recognize expressions	To investigate emotion recognition development in young children with ASD.
Tracy et al. (39)	Prospective cohort	Canada	ASD 29, HC 31	29	Mean age 12.25	Emotion recognition Overall Accuracy: ASD = 77%, TD = 76% (no significant difference). Pride Recognition: ASD = 88%, TD = 89% (better than basic emotions, *p* < 0.05). Fear and Contempt: Lowest recognition rates, not above chance. Response Times: No significant differences between groups.	ASD does not prevent emotion recognition	To determine if ASD prevents emotion recognition.

ASD, autism spectrum disorder; PWS, prader-willi syndrome; HC, healthy controls; ASD-ID, autism spectrum disorder with intellectual disabilities; FER, facial emotion recognition; FERT, facial emotion recognition task; FEEL, facial expressions of emotion labeling; FEFA, facial expression and feeling assessment; BERT, basic emotion recognition test; TD, typically developing; HFA, high-functioning autism; LFA, low-functioning autism; FC, fixation count; FDT, fixation duration time; SCZ, schizophrenia.

### Quality of included studies

The quality of the included studies was assessed using the Newcastle-Ottawa Scale for nonrandomized trials (Table 2). This standardized tool evaluates the methodological rigor of studies based on three domains: selection of study groups, comparability of groups, and outcome ascertainment. Of the 13 studies included in the systematic review, 12 achieved a perfect score of 9/9 on the Newcastle-Ottawa Scale (NOS). However, the meta-analysis was conducted on all 13 studies to ensure comprehensive results. Sensitivity analyses were performed to evaluate the impact of studies with lower NOS scores, confirming that their inclusion did not significantly affect the overall findings.

**Table 2:** Quality assessment using Newcastle-Ottawa scale.

Quality assessment of included studies									
Study ID	Selection				Comparability	Outcome			Total
	Is the case definition adequate?	Representativeness of cases	Selection of controls	Definition of Controls	Comparability of cases and controls on the basis of the design or analysis	Ascertainment of Outcome	Was follow-up long enough for outcomes to occur	Adequacy of follow up cohorts	
Perosanz (28)	1	1	1	1	2	1	1	1	9
Nagy (29)	1	1	1	1	2	1	1	1	9
Greco (30)	1	1	1	1	2	1	1	1	9
Hauschild (31)	1	1	1	1	2	1	1	1	9
Mazzoni (32)	1	1	1	1	2	1	1	1	9
He (33)	1	1	1	1	2	1	1	1	9
Liu (34)	1	1	1	1	2	1	1	1	9
Kuusikko-Gauffin (35)	1	1	1	1	2	1	1	1	9
Høyland (40)	1	1	1	1	2	1	1	1	9
Xavier (36)	1	1	1	1	2	1	1	1	9
Tell (37)	1	1	1	1	2	1	1	1	9
Rump (38)	1	1	1	1	2	1	1	1	9
Tracy (39)	1	1	0	1	2	1	1	1	8

### Meta-analysis results

#### Overall emotion recognition accuracy

**Figure 2:**
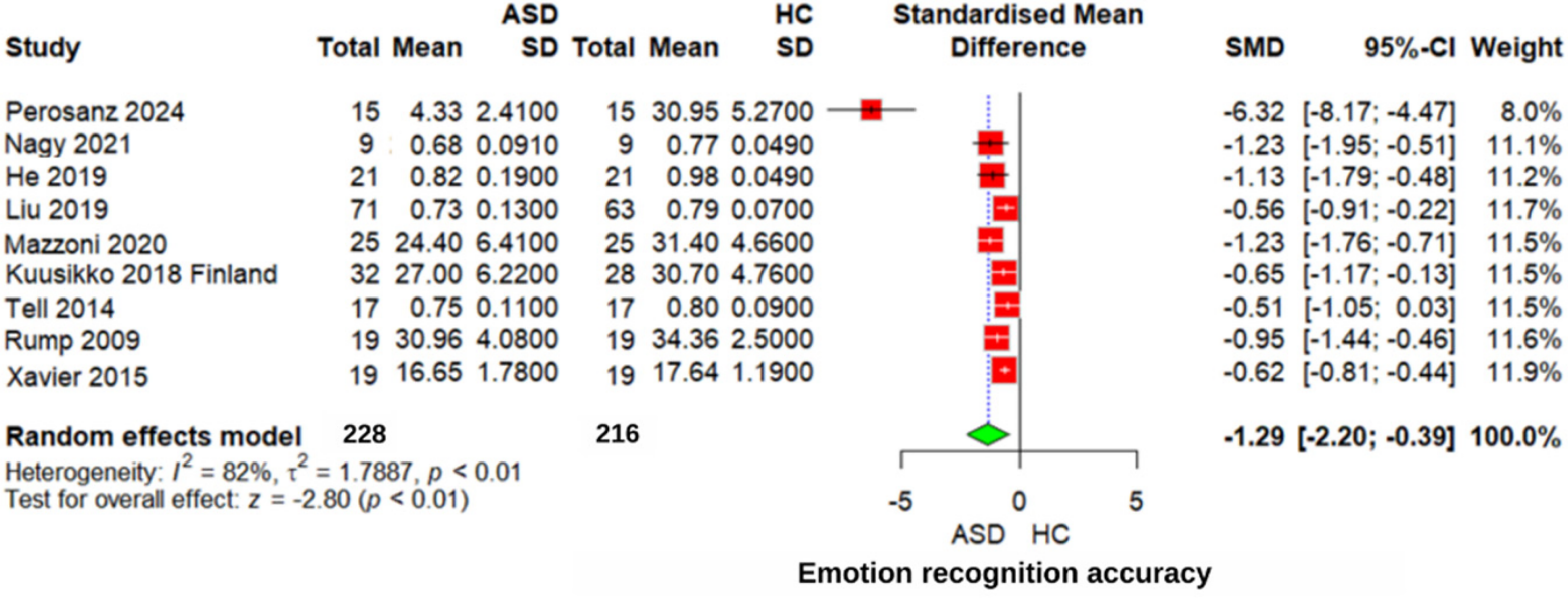
Forest plot for overall emotion recognition accuracy in ASD vs. Controls.

Individuals with ASD demonstrated significantly lower emotion recognition accuracy compared to both neurotypical individuals and those with neurodevelopmental disorders. A weighted meta-analysis yielded an effect size of SMD = −1.29 (95% CI: −2.20 to −0.39, *z* = −2.80, *p* < 0.01) for comparisons with neurotypical individuals, with substantial heterogeneity observed across studies (*I*^2^ = 82%, *τ*^2^ = 1.7887), indicating variability in participant characteristics and methodologies (Figure 2).

Comparisons with neurodevelopmental disorders revealed smaller but still significant deficits, with an effect size of SMD = −0.89 (95% CI: −1.23 to −0.55, *p* = 0.02). The heterogeneity for this comparison was moderate (*I*^2^ = 50%, *τ*^2^ = 0.4821), reflecting less variability than observed in comparisons with neurotypical individuals.

The overall emotion recognition accuracy of individuals with ASD was compared to that of healthy controls (HC) using a sensitivity analysis. A significant overall effect was found (*z* = −7.92, *p* < 0.01), with a standardized mean difference (SMD) of −0.77 (95% CI: −0.97 to −0.58), excluding Perosanz 2024 (28). This finding indicates that individuals with ASD performed significantly worse on emotion recognition tasks than HC did. Heterogeneity was low to moderate (*I*^2^ = 34%, *τ*^2^ = 0.0242), suggesting consistency across the studies.

### Emotion-specific analyses

#### Fear emotion recognition accuracy

**Figure 3:**
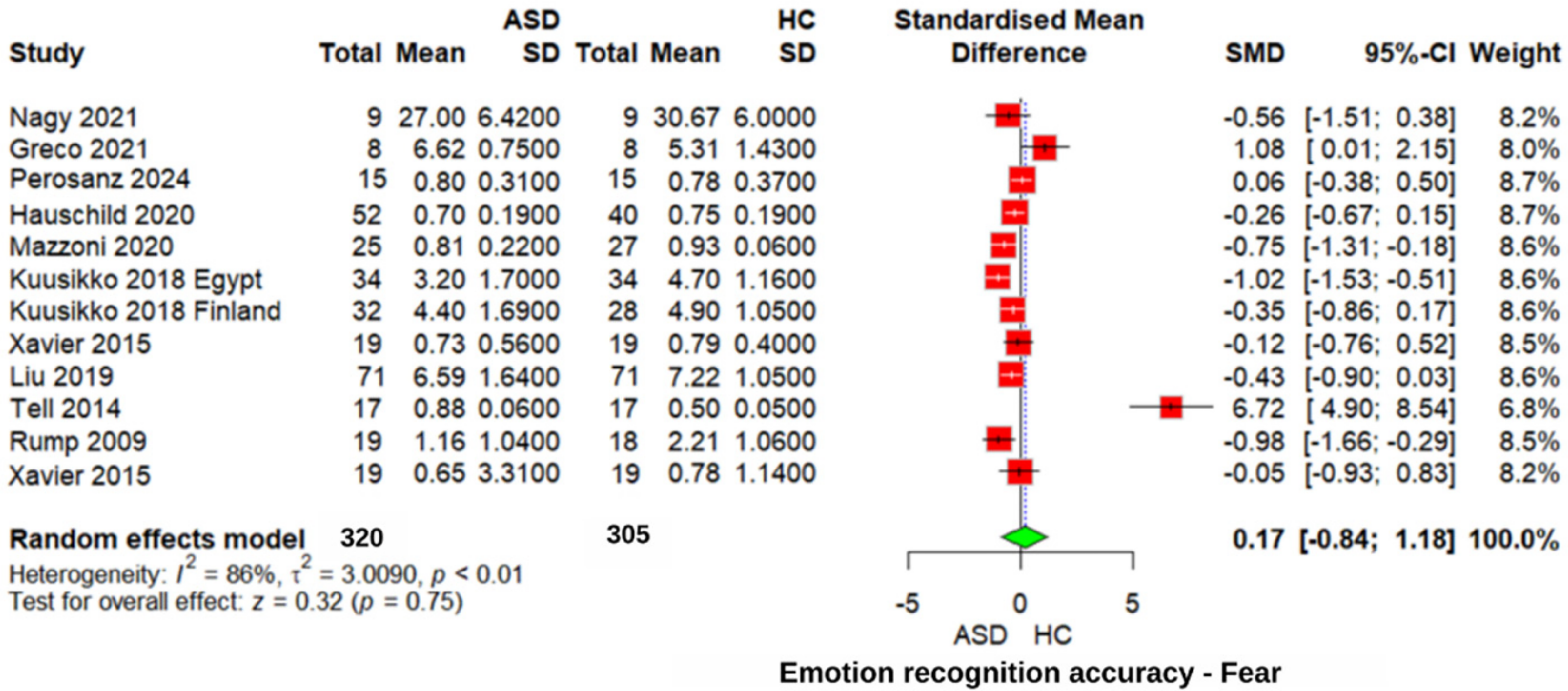
Forest plot for fear emotion recognition accuracy in ASD vs. Controls.

The meta-analysis of “fear” emotion recognition (Figure 3) found no significant difference between the ASD and neurotypical individuals' groups (*z* = 0.32, *p* = 0.75, SMD = 0.17, 95% CI: −0.84 1.18). Heterogeneity was very high (*I*^2^ = 86%, *τ*^2^ = 3.0090), indicating substantial variability across studies. Similarly, when compared to individuals with other neurodevelopmental disorders, no significant differences were observed for this emotion (SMD = 0.10, 95% CI: −0.67 to 0.87, *p* = 0.81). These findings suggest that recognizing “fear” emotions may not represent a specific area of difficulty for individuals with ASD.

#### Anger emotion recognition accuracy

**Figure 4:**
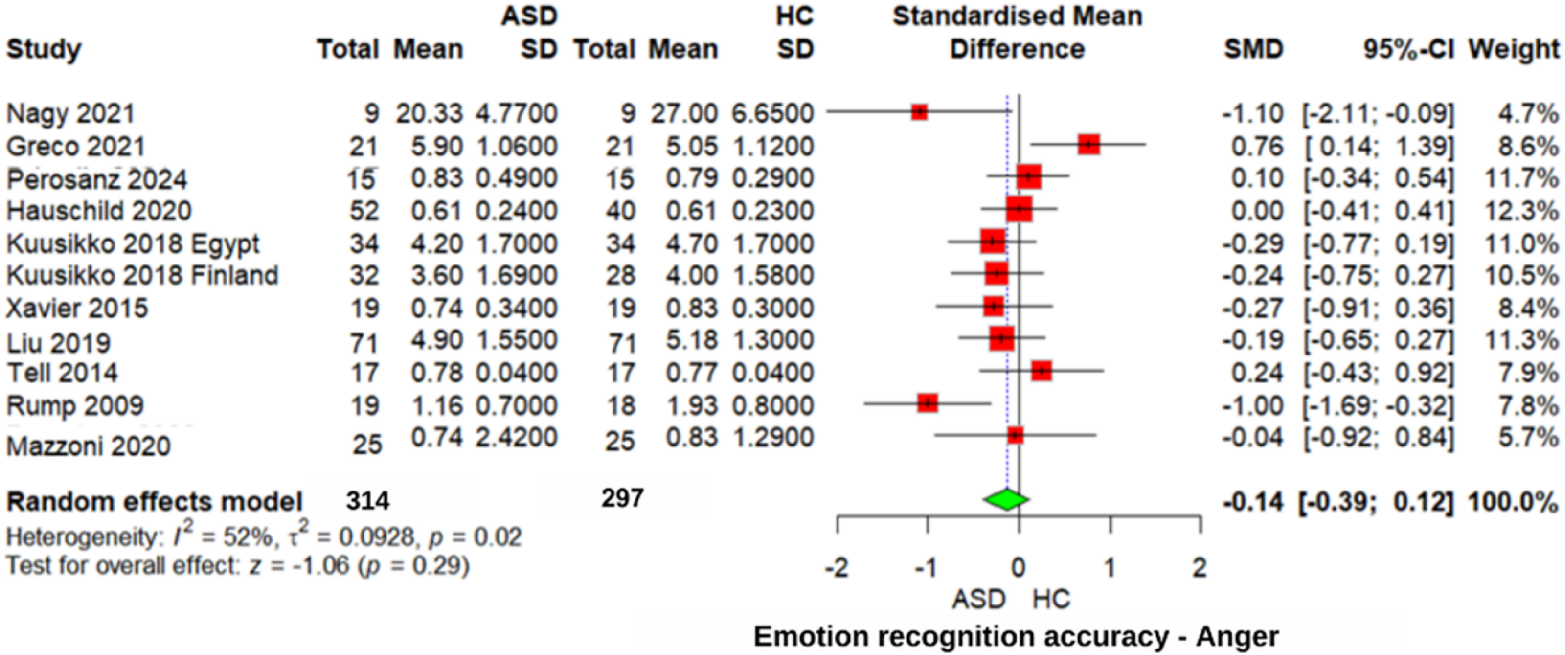
Forest plot for anger emotion recognition accuracy in ASD vs. Controls.

The analysis of “anger” emotion recognition (Figure 4) also found no significant difference (*z* = −1.06, *p* = 0.29, SMD = −0.14, 95% CI: −0.39 to 0.12), with moderate heterogeneity (*I*^2^ = 52%, *τ*^2^ = 0.0928). However, sensitivity analysis excluding Greco 2021 (30) showed a marginally significant effect (*z* = −1.96, *p* = 0.05, SMD = −0.19, 95% CI: −0.38 to 0.00), with lower heterogeneity (*I*^2^ = 30%). Similarly, comparisons between individuals with ASD and those with other neurodevelopmental disorders revealed no significant differences in recognizing “anger” emotions (SMD = −0.09, 95% CI: −0.45 to 0.26, *p* = 0.63). These findings indicate that recognizing “anger” emotions does not appear to be a distinguishing difficulty for individuals with ASD.

#### Disgust emotion recognition accuracy

**Figure 5:**
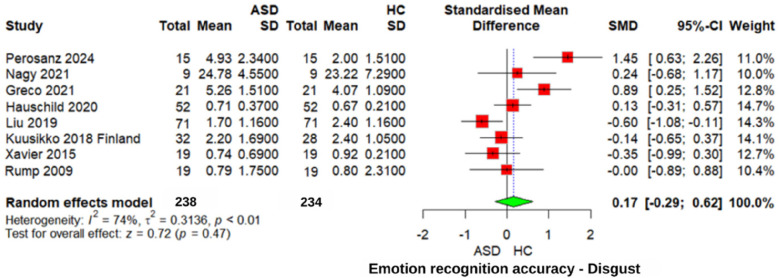
Forest plot for disgust emotion recognition accuracy in ASD vs. Controls.

In the “disgust” emotion recognition meta-analysis (Figure 5), no significant difference was observed (*z* = 0.72, *p* = 0.47, SMD = 0.17, 95% CI: −0.29 to 0.62). However, heterogeneity was high (*I*^2^ = 74%, *τ*^2^ = 0.3136), suggesting considerable variability across studies. Similarly, comparisons between individuals with ASD and those with other neurodevelopmental disorders yielded nonsignificant results for recognizing “disgust” emotions (SMD = 0.12, 95% CI: −0.34 to 0.59, *p* = 0.61). These findings suggest that difficulties in recognizing “disgust” emotions are not uniquely pronounced in individuals with ASD.

#### Happiness emotion recognition accuracy

**Figure 6:**
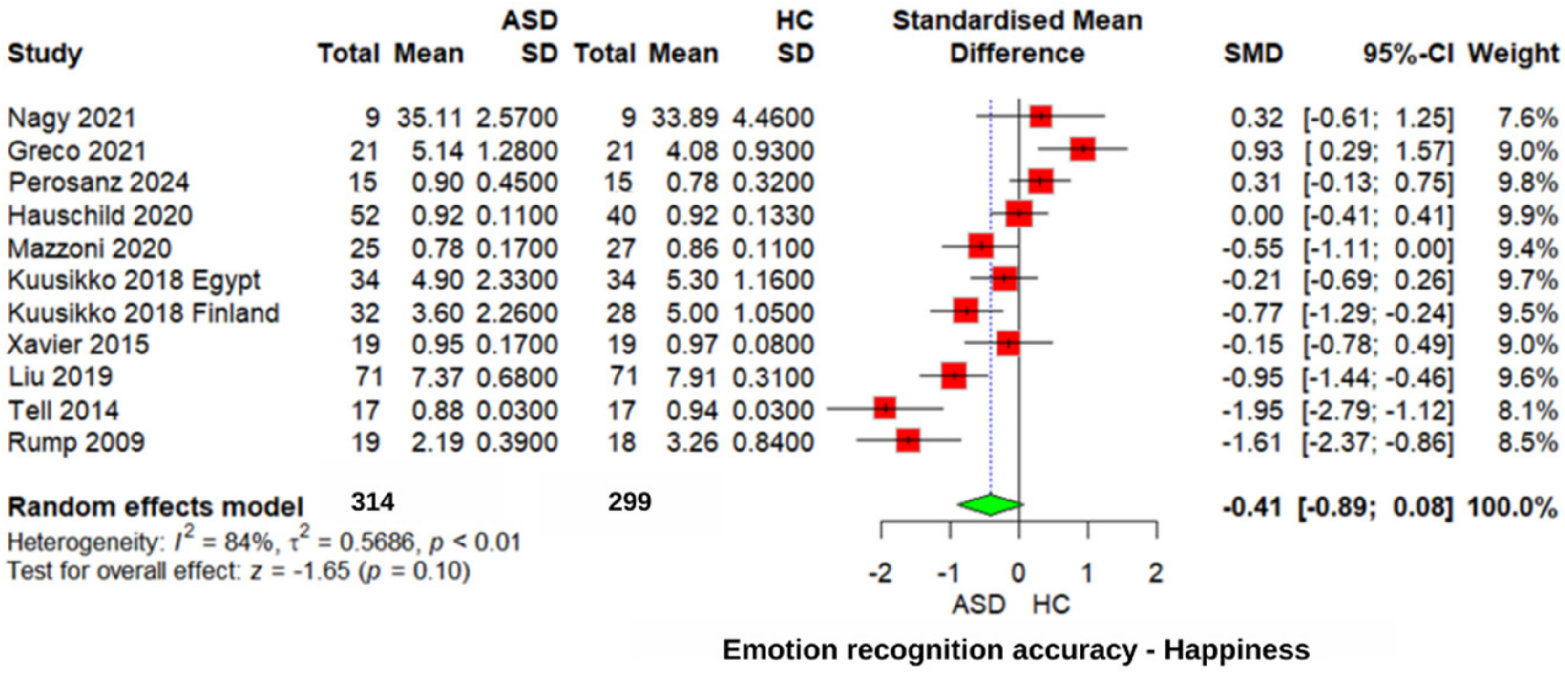
Forest plot for happiness emotion recognition accuracy in ASD vs. Controls.

The analysis of “happiness” emotion recognition (Figure 6) showed a non-significant trend toward poorer recognition in ASD patients (*z* = −1.65, *p* = 0.10, SMD = −0.41, 95% CI: −0.89 0.08). The heterogeneity was very high (*I*^2^ = 84%, *τ*^2^ = 0.5686), indicating substantial variability in the study findings. Similarly, comparisons between individuals with ASD and those with other neurodevelopmental disorders revealed no significant differences in recognizing “happiness” emotions (SMD = −0.25, 95% CI: −0.68 to 0.18, *p* = 0.26). These findings suggest that difficulties in recognizing “happiness” emotions may not be unique to ASD.

#### Sadness emotion recognition accuracy

**Figure 7:**
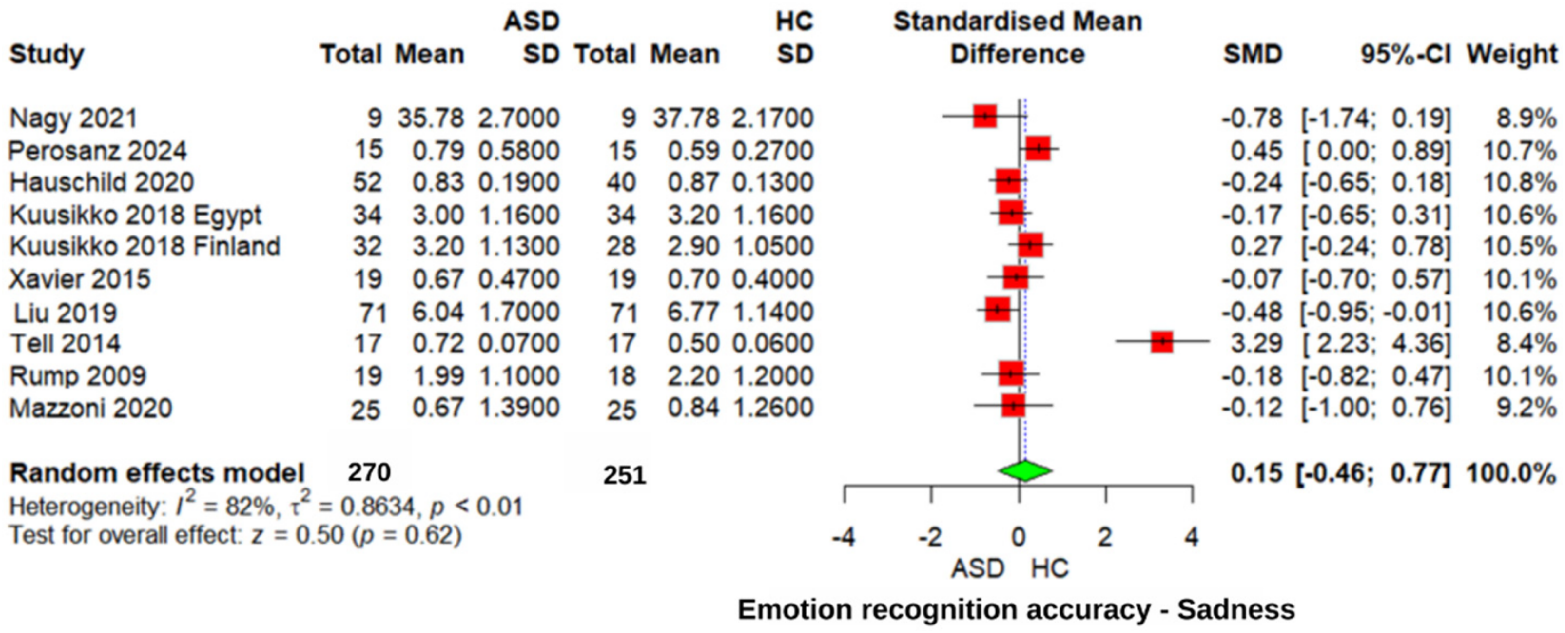
Forest plot for sadness emotion recognition accuracy in ASD vs. Controls.

For the recognition of “sadness” emotions, no significant difference was observed between the ASD and HC groups (*z* = 0.50, *p* = 0.62, SMD = 0.15, 95% CI: −0.46 0.77). The heterogeneity was high (*I*^2^ = 82%, *τ*^2^ = 0.8634), reflecting greater consistency across studies (Figure 7). Similarly, comparisons between individuals with ASD and those with other neurodevelopmental disorders revealed non-significant differences in recognizing “sadness” emotions (SMD = 0.05, 95% CI: −0.49 to 0.59, *p* = 0.85).

A sensitivity analysis excluding one study [Tell 2014 (24)] yielded slightly different results, with an effect size of SMD = −0.10 (95% CI: −0.34 to 0.14, *z* = −0.79, *p* = 0.43), and heterogeneity was reduced to a mild level (*I*^2^ = 38%, *τ*^2^ = 0.0511). These findings suggest that recognition of “sadness” emotions does not represent a significant challenge specific to individuals with ASD and remains consistent across study designs.

#### Surprise emotion recognition accuracy

**Figure 8:**
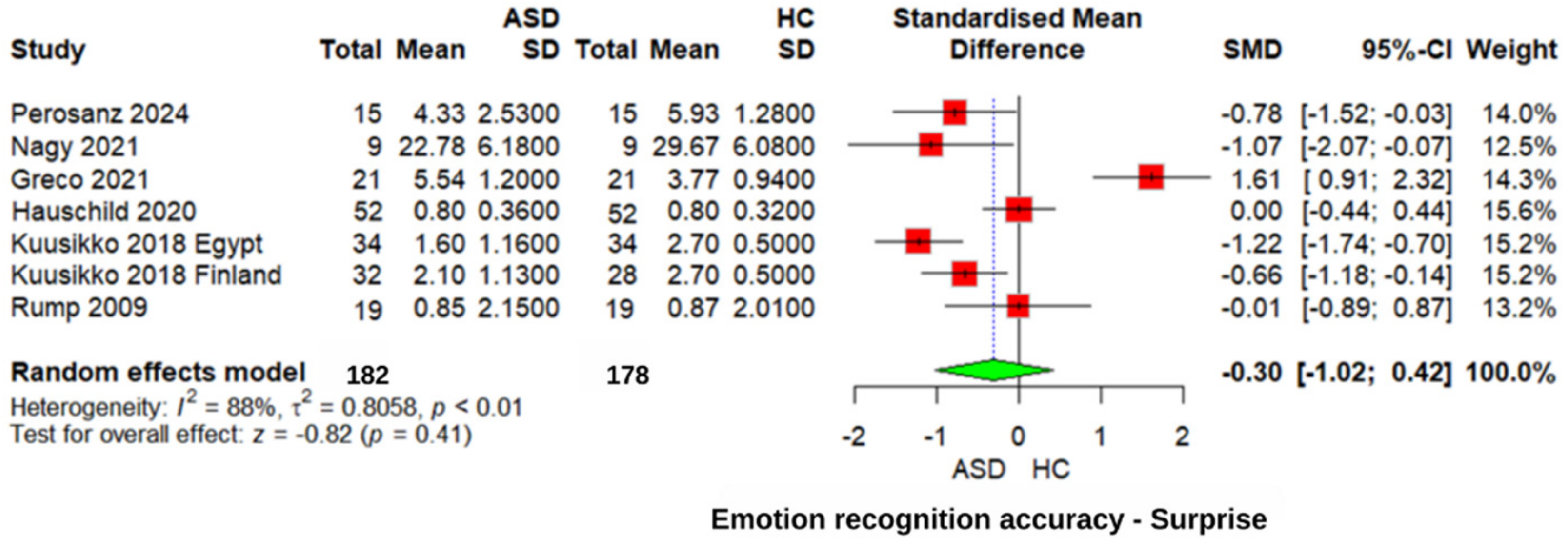
Forest plot for surprise emotion recognition accuracy in ASD vs. Controls.

For the recognition of “Surprise” emotions (Figure 8), no significant difference was observed between the ASD and HC groups (*z* = 0.82, *p* = 0.41, SMD = −0.30, 95% CI: −1.02 0.42). The heterogeneity was high (*I*^2^ = 88%, *τ*^2^ = 0.8058), reflecting greater consistency across studies. Similarly, comparisons with individuals with other neurodevelopmental disorders showed no significant differences in recognizing “surprise” emotions (SMD = −0.21, 95% CI: −0.87 to 0.46, *p* = 0.54). These findings suggest that recognition of “surprise” emotions is not specifically impaired in individuals with ASD.

### Response time meta-analyses

#### Overall response time

For overall response times, individuals with ASD demonstrated significantly longer response times compared to neurotypical individuals, with a standardized mean difference (SMD) of 0.50 (95% CI: 0.36–0.63, *p* < 0.01), indicating that they took more time to process emotional expressions compared to HC. Heterogeneity across studies was low to moderate (*I*^2^ = 37%, *τ*^2^ = 0.0229), indicating relative consistency in this finding.

In contrast, when comparing individuals with ASD to those with other neurodevelopmental disorders, no significant differences in response times were observed (SMD = 0.12, 95% CI: −0.08 to 0.32, *p* = 0.15). Heterogeneity for this comparison was moderate (*I*^2^ = 40%), reflecting some variability in study results. These findings suggest that prolonged response times in individuals with ASD may be more distinct when compared to neurotypical individuals than to those with other neurodevelopmental disorders.

#### Fear emotion response time

For “fear” emotion recognition response times, no significant difference was observed among individuals with autism spectrum disorder compared to either neurotypical individuals or those with other neurodevelopmental disorders (SMD = 0.33, 95% CI: −0.05 to 0.71, *z* = 1.69, *p* = 0.09). Moderate heterogeneity was observed across studies (*I*^2^ = 50%, *τ*^2^ = 0.0944), reflecting some variability in the findings.

#### Anger emotion response time

The analysis of response times for recognizing “anger” emotions showed no significant effect were found across the groups, with an SMD of 0.33 (95% CI: −0.05 to 0.71, *z* = 1.69, *p* = 0.09), with moderate heterogeneity (*I*^2^ = 50%, *τ*^2^ = 0.0944) suggesting consistency in the results.

#### Happiness emotion response time

No significant difference was observed in response times for recognizing “happiness” emotions when comparing individuals with ASD to either neurotypical individuals or those with neurodevelopmental disorders. The SMD was 0.16 (95% CI: −0.28−0.61, *z* = 0.72, *p* = 0.47), although heterogeneity was substantial (*I*^2^ = 64%, *τ*^2^ = 0.1587), indicating greater variability in study methodologies and populations.

## Discussion

### Summary of findings

This meta-analysis synthesized findings from 13 studies to evaluate emotion recognition accuracy and response time in individuals with ASD compared with control groups, including healthy controls (HC) and individuals with other neurodevelopmental conditions (NDDs). Results revealed significant impairments in overall emotion recognition accuracy among individuals with ASD compared to both comparator groups, with larger deficits observed when compared to TD individuals (SMD = −1.29, *p* < 0.01) than to NDDs (SMD = −0.89, *p* = 0.02). Additionally, individuals with ASD exhibited prolonged response times relative to TD individuals (SMD = 0.50, *p* < 0.01), though no significant differences were found when compared to NDDs. These findings highlight pervasive challenges in social cognition among individuals with ASD, extending across both accuracy and processing speed.

However, when examining emotion-specific recognition, namely, fear, anger, disgust, happiness, sadness, and surprise, the analyses did not consistently reveal significant differences between the ASD and control groups, although substantial heterogeneity was noted across these specific emotions. Sensitivity analyses, which excluded studies contributing disproportionately to heterogeneity, affirmed the robustness of the overall accuracy deficit (SMD = −0.77, *p* < 0.01) and highlighted more consistent findings across studies when certain outliers were removed.

### Interpretation of results

The observed deficits in emotion recognition accuracy and prolonged response times in individuals with ASD underscore the social communication challenges associated with this condition. Emotion recognition is integral to successful social interactions, and impairments in this domain may contribute to difficulties in forming and maintaining relationships. The significant reduction in accuracy suggests that individuals with ASD may struggle to interpret and respond to emotional cues, potentially leading to the social isolation and interpersonal difficulties frequently reported in this population.

Prolonged response times among individuals with ASD indicate not only difficulties in accurately identifying emotions, but also inefficiencies in processing emotional stimuli. This delay can impede real-time social interactions, where the rapid interpretation of emotional states is often critical for appropriate and timely responses. The combination of lower accuracy and prolonged processing times may lead to social communication challenges, reinforcing the social deficit characteristic of ASD. The non-significant differences in response times between ASD and NDD groups suggest that delayed processing may be a broader characteristic of neurodevelopmental disorders.

The lack of significant differences in emotion-specific recognition across most emotions suggests that emotion recognition impairments in ASD patients may not be uniformly distributed across all emotional categories. However, the high heterogeneity in these analyses indicates that variability in study methodologies, participant characteristics, and assessment tools may obscure consistent patterns, making it difficult to draw definitive conclusions regarding specific emotional deficits. Cultural differences, as noted in some included studies, may influence emotion recognition abilities and error patterns, further contributing to the variability in the findings.

### Comparison with previous literature

Palmer et al. (41) investigated the impact of emotional valence on emotion recognition in adolescents with ASD using the Reading the Mind in the Eyes Task (RMET), developed by Baron-Cohen et al. (15). They found that individuals with ASD made more errors on positive and negative valence items, but not on neutral ones, with larger discrepancies observed in the adult version of the RMET than in the child version. Additionally, both emotional valence and language complexity influenced performance disparities in ASD patients. These findings are consistent with those of the current meta-analysis, highlighting how specific emotional contexts and task complexities exacerbate emotion recognition deficits in ASD.

Ozbek et al. (42) conducted a systematic review and meta-analysis to compare social and nonsocial cognition in patients with schizophrenia and ASD. They reported that schizophrenia is associated with more severe non-social cognitive impairments, particularly in fluency and processing speed, whereas ASD is linked to more pronounced social cognitive deficits when matched for non-social cognition or reasoning and problem-solving abilities. This differentiation underscores the distinct cognitive profiles of ASD compared with other developmental disorders, emphasizing the need for targeted interventions addressing ASD-specific cognitive challenges.

Griffin et al. (23) examined the face inversion effect in ASD using behavioral and neural measures. This study demonstrated that autistic individuals exhibit a reduced face inversion effect compared to neurotypical individuals, suggesting diminished specialization in the face processing system. This attenuated inversion effect was more evident in emotion recognition tasks and behavioral measures, supporting the findings of the current meta-analysis on overall emotion recognition deficits in ASD.

The mixed results of emotion-specific analyses align with studies indicating that emotions such as fear and anger may be particularly challenging for individuals with ASD [Perosanz (28)]. However, the lack of consistent significant differences across all emotions in this meta-analysis suggests that emotion recognition impairments in ASD are influenced by multiple interacting factors beyond emotion type.

### Sources of heterogeneity

The substantial heterogeneity observed in overall emotion recognition accuracy (*I*^2^ = 82%) and across specific emotions indicates considerable variability in the study outcomes. This heterogeneity can be attributed to several factors, including variations in participant characteristics such as age ranges, intellectual abilities, and the presence of comorbid conditions across studies. Differences in study design, diagnostic criteria, and emotion recognition assessment tools also introduce methodological variability that affects comparability across studies. Additionally, cultural and linguistic factors may play a role in emotion recognition abilities, influencing results in diverse populations.

For example, studies conducted in different cultural contexts may use emotion recognition tasks that are culturally biased or interpret emotional expressions differently, contributing to inconsistent findings. Variations in the severity of ASD and the presence of co-occurring conditions, such as ADHD or intellectual disabilities, further complicate the synthesis of results. Moreover, the use of diverse assessment tools, such as the Reading the Mind in the Eyes Test (RMET)and facial emotion recognition tasks, are likely to capture different aspects of social cognition, thereby increasing variability.

## Implications for theory, intervention, and future research

The findings of this meta-analysis have significant implications for theoretical models of social cognition in ASD and practical intervention strategies. The robust deficits in overall emotion recognition accuracy and prolonged response times support theoretical frameworks that emphasize impairments in social information processing and emotional empathy in ASD patients. These deficits likely contribute to the broader social communication challenges observed in individuals with ASD, reinforcing the need for interventions targeting both the accuracy and efficiency of emotion recognition.

From a practical standpoint, these results highlight the importance of incorporating emotion recognition training into therapeutic programs for ASD. Such interventions could focus on enhancing the ability to accurately identify and interpret a wide range of emotional expressions as well as improve the speed of processing emotional cues to facilitate more fluid and naturalistic social interactions. Additionally, the variability in emotion-specific findings suggests that personalized intervention approaches may be necessary to address the specific emotional processing challenges faced by individuals with ASD. For example, targeted training to recognize emotions with higher difficulty, such as fear and anger, could be beneficial. Furthermore, integrating findings from comparative studies, such as Ozbek et al. (42), can inform the development of specialized cognitive training programs that address both social and nonsocial cognitive deficits unique to ASD.

Insights from Griffin et al. (23) also suggest that interventions may need to account for the reduced specialization in face processing among individuals with ASD. Incorporating strategies that enhance the ability to process facial orientation, and emotional complexity can further improve emotion recognition. Additionally, leveraging neurobiological findings to inform intervention design may enhance the effectiveness of therapeutic programs aimed at mitigating emotion-recognition deficits.

Future research should aim to mitigate the heterogeneity identified in this meta-analysis by adopting more standardized methodologies for assessing emotion recognition in ASD patients. Using consistent diagnostic criteria, comparable emotion recognition tasks, and homogeneous participant samples regarding age, IQ, and comorbid conditions can enhance the comparability of studies. Longitudinal research is needed to explore the developmental aspects of emotion recognition deficits and their influence on social functioning over time.

Additionally, exploring the neural underpinnings of emotion recognition deficits in ASD through neuroimaging studies can provide deeper insight into the cognitive processes involved. Investigating the role of contextual information integration and examining the impact of cultural factors on emotion recognition abilities can further elucidate the complexities of social cognition in ASD patients. Developing and evaluating targeted interventions aimed at improving both the accuracy and speed of emotion recognition are crucial. Assessing the efficacy of such interventions in diverse populations and settings is essential for translating research findings into practical applications that can enhance social communication skills in individuals with ASD. Moreover, focusing on specific emotions that may be particularly challenging for individuals with ASD, such as fear and anger, could lead to the development of tailored therapeutic strategies addressing these areas.

### Limitations

Several limitations should be considered when interpreting the results of this meta-analysis. The considerable variability across studies limits the generalizability of the findings and suggests that pooled effect sizes may not accurately represent the true effect in all contexts. Differences in study design, participant demographics, diagnostic criteria, and emotion recognition tasks contribute to this variability and make it challenging to draw definitive conclusions. Additionally, most of the included studies employed cross-sectional designs, which preclude the assessment of developmental trajectories and causal relationships between ASD and emotion recognition deficits.

Publication bias is another potential limitation, as studies with significant findings are more likely to be published, potentially skewing the meta-analysis results. Although publication bias was assessed using funnel plots and Egger's test, the analyses were not explicitly reported. Future meta-analyses should ensure the inclusion of such assessments to provide a more comprehensive evaluation of evidence. Furthermore, the exclusion of studies that did not provide sufficient data to calculate effect sizes may have led to the omission of relevant research, potentially affecting the overall findings. The reliance on published studies also meant that unpublished data, which might offer valuable insights, were not considered.

## Conclusion

This systematic review with a meta-analysis provides compelling evidence for significant deficits in overall emotion recognition accuracy and prolonged response times in individuals with autism spectrum disorder compared to neurotypical individuals and those with other neurodevelopmental disorders. While emotion-specific findings were less consistent, the overarching results underscore the critical role of emotion recognition impairments in the social challenges faced by individuals with ASD. Addressing these deficits through targeted interventions and standardized research approaches is essential for improving social communication skills and quality of life for individuals on the autism spectrum.

## Data Availability

The original contributions presented in the study are included in the article/Supplementary Material, further inquiries can be directed to the corresponding author.
